# Partial substitution of chemical fertilizer with organic fertilizer: a promising circular economy approach for improvement soil physical and chemical properties and sustainable crop yields

**DOI:** 10.3389/fpls.2025.1565081

**Published:** 2025-05-20

**Authors:** Qian Chen, Junhong Xie, Lingling Li, Khuram Shehzad Khan, Linlin Wang, Lei Chang, Changliang Du

**Affiliations:** ^1^ State Key Laboratory of Aridland Crop Science, Gansu Agricultural University, Lanzhou, China; ^2^ College of Agronomy, Gansu Agricultural University, Lanzhou, China

**Keywords:** organic fertilizer, soil organic carbon fraction, maize, sustainable yield, semi-arid loess plateau, economic benefit

## Abstract

**Introduction:**

Food security faces multiple challenges, and increasing crop yields is an effective way to address this issue. Replacing chemical fertilizers (CFs) with organic fertilizers can affect soil nutrient cycling and hence crop yields, with changes in organic carbon content being an important way in which soil nutrient content affects crop production. However, the dynamics of the effect of organic fertilizer substitution on soil organic carbon and the mechanism by which it further contributes to yield formation are not clear.

**Methods:**

To this end, a 2 - year maize field experiment (2019-2020) was conducted to study the effect of organic substitution on soil properties, organic carbon fractions, and maize yields. Six treatments were applied: no fertilizer (CK), CF, and four different organic substitution rates (50%, 37.5%, 25%, and 12.5%), denoted by (50% OF), (37.5% OF), (25% OF), and (12.5% OF), respectively. Fully film - mulched double ridge-furrow technology was used to optimize water retention and soil temperature.

**Results:**

Results demonstrated that 12.5% OF reduced water consumption by 1.40% during critical maize growth stages compared to CF. It also increased 0-30 cm total phosphorus (TP) by 15.09%, soil porosity by 4.82%, and available phosphorous (AP) by 34.81% at harvest, respectively, compared with CK of 2 years average. Partial substitution of CF with organic fertilizer led to a significant increase in soil organic carbon (SOC) and fractions through improvement in physicochemical properties. The 12.5% OF at 0-30 cm soil layer significantly increased easily oxidizable organic carbon (EOC) by 33.23%, SOC by 2.18%, and particulate organic carbon (POC) by 6.64% compared to CF, respectively. At 10-30 cm, 37.5% OF increased microbial biomass carbon (MBC) by 9.90% and hot water-soluble carbon (HOC) by 6.90% compared to CF. Under 12.5% OF, an EOC increased by 13.20% at 0-5 cm, while dissolved organic carbon (DOC) and light fraction organic carbon (LFOC) rose by 18.65% and 37.13% at 0-10 cm, respectively. Interestingly, the 12.5% OF boosted grain yields by 6.60% and biomass by 4.59% compared to CF, and by 213.02% and 208.13% compared to CK. Water use efficiency (WUE) increased by 11.43% and 153.27% under CK and CF treatment, respectively. Randomized forest analysis highlighted that increases in soil MBC, HOC, and DOC content were critical for maize yield improvement.

**Discussion:**

In summary, 12.5% OF and 37.5% OF can increased MBC, HOC, and DOC content by increasing soil porosity, TP and AP content and decreasing soil water depletion, thereby increasing crop yield. Compared to 37.5% OF, 12.5% OF was more environmentally sustainable, increased crop yields, and increase economic benefits. This provided a theoretical basis for partial substitution of CF with organic fertilizer to improve soil health and crop yield. The present study showed that 12.5% OF (200 kg hm^-2^ of N) was a suitable cropping pattern in the region and was recommended for wider use in the region.

## 1 Introduction

Food security faces multiple challenges, including population growth, climate change, land degradation, and water scarcity. The combination of these factors has made the issue of food security more complex and urgent (He et al., 2021; Prăvălie, 2021; Du et al., 2023). Furthermore, the sustainable production of agricultural crops gained much attention in maintaining national food security (Lamptey et al., 2017). Maize is an important food and forage crop in China and accounts for 23% of the total annual global production (Xu et al., 2023). Meanwhile, most rainfed areas in central Gansu Province grow maize crops, and the heavy use of chemical fertilizers (CFs) has ensured the production of maize in the region. Over the past 30 years, CF has been widely used throughout the world, but especially in China, to ensure high crop yields sufficient to satisfy the demands of growing populations (Stewart and Roberts, 2012; Li et al., 2022). However, excessive and prolonged use of CFs degrades soil quality and creates environmental issues (Iqbal et al., 2022). The long-term application of high doses of CFs may also affect the soil carbon pool; therefore, fertilizer inputs (organic and inorganic) need to be optimized by carbon (C) amendments (Khan et al., 2023). In recent reports, the application of organic amendments (manure, straw, etc.) has been shown to result in improved soil physical and chemical properties, enhanced soil carbon contents, increased crop yields, and the effective enhancement of biogeochemical nutrient cycling in soil systems (Li et al., 2020b; Khan et al., 2022).

Organic fertilizers could improve the physical structure of the soil, such as reducing soil bulk density (SB) and improving soil aeration and water permeability (Ren et al., 2021). In addition, related studies have shown that partial substitution of CF with organic fertilizer can reduce soil acidification, increase soil organic carbon (SOC) and nutrient content, and thus improve soil physicochemical properties (Song et al., 2022). In previous studies, partial substitution of CF with organic fertilizer improved soil nitrogen, phosphorus, and potassium content; increased SOC pool stability; and ensured soil nutrient utilization compared to chemical or organic fertilizers alone (Li et al., 2020b; Khan et al., 2022; Zhang et al., 2022; Yang and Zhang, 2023b).

Soil physicochemical properties have important effects on soil carbon content and stability (Jiménez-González et al., 2020). To fully evaluate the stability of soil carbon pools, it is important to understand the relationship between soil physicochemical factors and soil carbon fractions such as EOC, LFOC, and MBC (Manna et al., 2005; Li et al., 2018; Tang et al., 2020). SOC is the largest C pool and is represented as the soil quality index in terrestrial ecosystems. It is also known as an important facet of fertility and productivity by promoting the physicochemical and biological characteristics (Sahoo et al., 2019; Terra et al., 2019; Bai et al., 2020; Ding et al., 2023). It has been found that SOC is higher in acidic soils and lower in alkaline soils (Jiménez-González et al., 2020). A meta-analysis also found that a continuous decline in soil pH and a decrease in soil aggregate organic carbon promoted POC accumulation (Lu et al., 2021). It is also found that pH is significantly correlated negatively with MBC (Peng et al., 2019). This shows that soil pH is an important factor affecting soil C. Soil water content is also an important factor affecting the soil C pool. Research has shown that the soil water content is correlated with soil C (Keller et al., 2021). Moderately moist soils favor the accumulation of SOC, while dry or excessively moist soils may lead to accelerated organic carbon decomposition (Li et al., 2020c). Increased soil total nitrogen (TN) content contributes to the accumulation of soil organic matter (SOM), thus favoring carbon storage (Soria et al., 2021). For example, an increase in TN content is associated with an increase in reactive SOC content and a decrease in inert SOC content (Li et al., 2020a). Ammonium-N (NH_4_^+^-N) is a direct nitrogen source used by microorganisms to synthesize proteins and other cellular components. An increase in NH_4_^+^-N content can stimulate microbial activity. This enhanced activity may accelerate the decomposition of organic carbon, leading to a reduction in SOC content (Li et al., 2019). In humid areas, NH_4_^+^-N tends to leach down through the soil, leading to SOC loss. Available phosphorous (AP) provides the phosphorus needed for microbial decomposition, so increased AP levels accelerate SOC decomposition (Shi et al., 2020). Studies have shown that soil total phosphorus (TP) content and SOC are also usually positively correlated (Zhao et al., 2021). In conclusion, changes in soil physical and chemical properties have a complex impact on SOC and carbon fractions, which need to be analyzed specifically according to soil types, climatic conditions, and management measures.

Increased SOC can improve SOM content and enhance the creation of the SOC pool, thereby ultimately improving soil quality and promoting food production (Raiesi, 2021). Among different SOC pools, soil active carbon pools are used to indicate early changes in SOM and influence the stability of soil carbon pools (Chen et al., 2022). Further, the relatively small proportion of these carbon pools, rapid turnover, and high sensitivity to environmental changes (Wang et al., 2015; Yang et al., 2023a). It is documented that organic fertilizers can provide additional C sources to the soil to improve soil nutrients and fertilizer use efficiency, which in turn increases crop yields (Yang et al., 2021; Oyetunji et al., 2022). According to a long-term fertilization study by He et al. (2022), it was found that an increase in available nutrients and SOC fractions improved soil quality and wheat yields with the application of chemical and organic fertilizers. Thus, to optimize fertilization approaches and promote agricultural ecosystem functions, it is crucial to enhance the understanding of carbon pools by substituting CF with organic fertilizer.

In dry cropland areas, the fully film mulched double ridge-furrow is a reliable cultivation technology that provides soil water content conservation services (Xie et al., 2020). It collects rainfall in the furrow through the ridge, thereby promoting the absorption and utilization of water by the crop, which in turn increases yield and efficiency (Xie et al., 2024). However, the prevailing agricultural practice of employing minimal or no organic fertilizer and a substantial quantity of CF is a common occurrence (Li et al., 2022). The use of organic fertilizers may change the nature and environment of the soil, and there is a need to further explore the effects of organic fertilizers as an alternative to CF management practices on the physicochemical properties of agricultural soils.

Previous studies indicate that the improvement of soil physicochemical properties is an important reason for organic fertilizer substitution for CFs to promote high crop yields, but its dynamic effect on soil physical and chemical properties is not clear. At the same time, soil physicochemical properties are closely related to soil C pools, so the specific relationship between them requires further investigation. Additionally, although increases in soil C pools directly influence crop yields, the key soil C indicators that most impact yields in semi-arid areas need to be identified and clarified. Thus, it is hypothesized that partial substitution of CF with organic fertilizer will improve soil nutrient availability, result in an increase in the accumulation of SOC sources, and thus promote growth and yield of maize crops in dry farmlands. The following are the main objectives of this research: (1) the effect of partial organic fertilizer replacement on soil physicochemical properties and soil active organic carbon fractions, coupling the relationship between the two to analyze the key factors affecting maize yield, (2) the impact of partial organic fertilizer substitution on maize yield and WUE, and (3) to evaluate the optimum N application dose for soil N fertility management to improve the yield efficiency of maize sown in the fully film-mulched double ridge-furrow in semi-arid areas. Partial substitution of CF with organic fertilizer and fully film mulched double ridge-furrow technology not only showed that it offers new insights for farmers on optimal fertilizer application but also provides a theoretical basis for improving soil health and crop yields in dry farming systems.

## 2 Materials and methods

### 2.1 Test site

The test site is situated at the Rainfed Agricultural Experimental Station of Gansu Agricultural University, in Dingxi City, Gansu Province, China. Its latitude and longitude are 35°28′N, 104°44′E, and the altitude is 1971 m. And the site is located in the south of central Gansu province, within a temperate semi-arid region. Average elevation 2000 m, average annual solar radiation 592.85 kJ cm^−2^, sunshine hours 2476.6h, average annual temperature 6.4°C, with an accumulated temperature above 0 degrees Celsius of 2933.5°C, and an accumulated temperature above 10 degrees Celsius of 2239.1°C, and the frost-free period 140 d. The annual average rainfall is 399.3 mm, with a dryness index of 2.53. This is a typical semi-arid rainfed agricultural area. The soil type is loess with a deep soil layer and a homogenized texture. The average SB in the 0–20 cm soil layer is 1.17 g cm^−3^, with a wilting moisture content of 7.3%, a saturated moisture content of 28.6%, a pH value of 8.36, an organic matter content of 11.92 g kg^−1^, a total N of 0.78 g kg^−1^, and a total P of 1.81 g kg^−1^.

### 2.2 Experimental design

**Figure 1:**
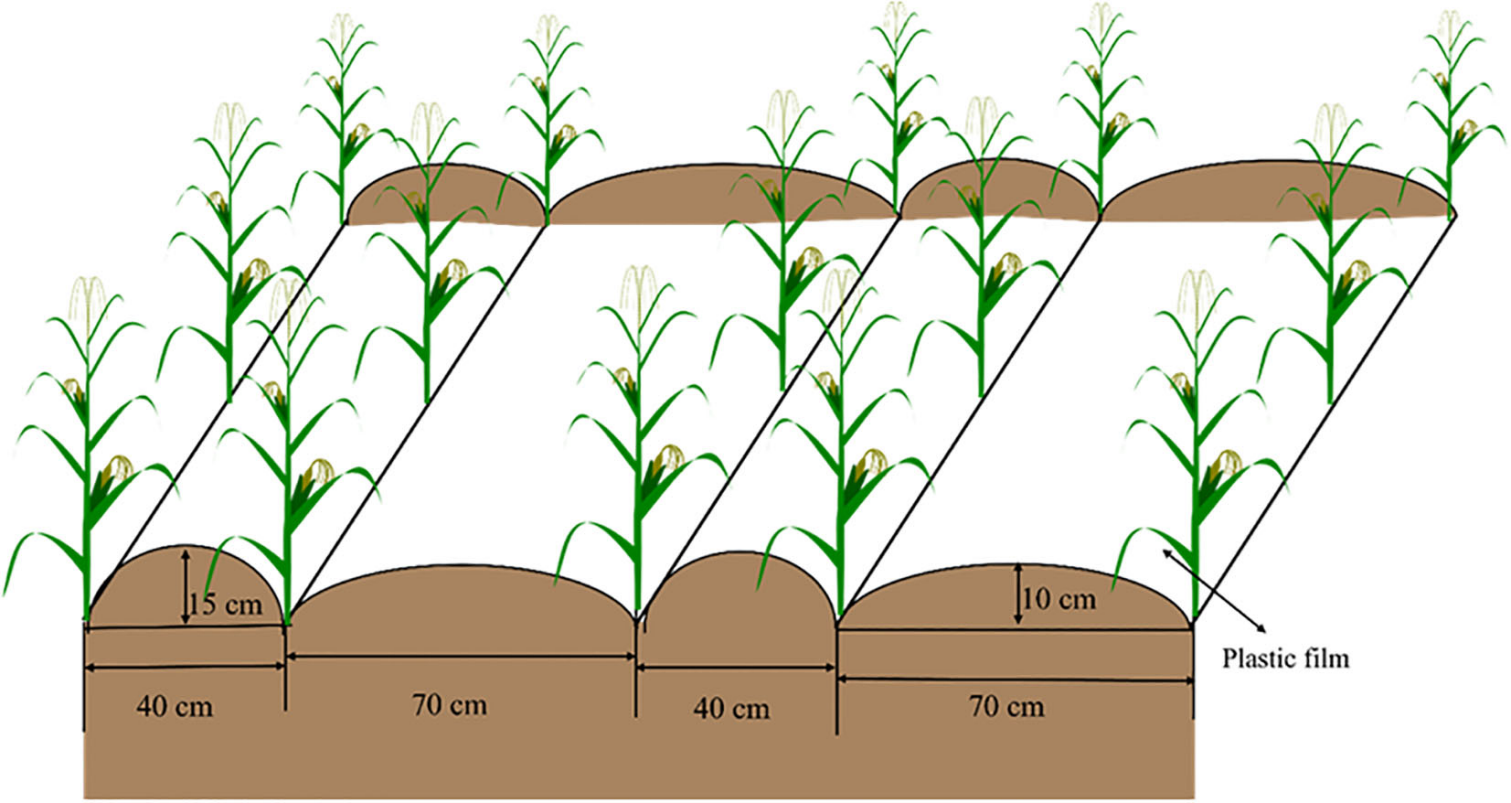
Graph of the whole film of double ridge furrow planting technology.

The objective of the field study was to cultivate a maize crop (April 2019–October 2020). Before maize sowing, the whole film of double ridge furrow planting technology (**Figure 1**) was used. Under the condition of equal N dose rate (200 kg ha^−1^), a randomized block design was employed, with six treatments, three replicates, and 18 plots. Six different ratios of organic fertilizer to fertilizer were set up for each plot, and the total area of the plot was 37.4 m^2^ (8.5 m× 4.4 m). Further, the fertilization application pattern was mentioned in **Table 1**.

**Table 1:** Fertilizer inputs under different treatments.

Code	Treatment	Ground fertilizer						Jointing fertilizer	Bell mouth fertilizer
		Organic fertilizer			Fertilizer			Fertilizer	
		N	P_2_O_5_	K_2_O	N	P_2_O_5_	K_2_O	N	
CF	100% fertilizer	0.0	0.0	0.0	100.0	150.0	10.6	60.0	40.0
50%OF	50% fertilizer +50% organic fertilizer	50.0	15.2	10.6	50.0	134.8	0.0	60.0	40.0
37.5%OF	62.5% fertilizer +37.5% organic fertilizer	37.5	11.4	8.0	62.5	138.6	2.7	60.0	40.0
25%OF	75% fertilizer +25% organic fertilizer	25.0	7.6	5.3	25.0	142.4	5.3	60.0	40.0
12.5%OF	87.5% fertilizer +12.5% organic fertilizer	12.5	3.8	2.7	87.5	146.2	8.0	60.0	40.0
CK	No fertilization	0.0	0.0	0.0	0.0	0.0	0.0	0.0	0.0

The commercial organic fertilizer (cow dung-based) was received by Gansu Hongyuan Biological Technology Corporation, Limited. The product’s chemical composition was as follows: total organic matter content was >46%, and the contents of N, P_2_O_5,_ and K_2_O were 3.3%, 1.0%, and 0.7%, respectively. The fertilization rate was 200 kg ha^−1^ of pure N, provided by urea (containing 46% N) and commercial organic fertilizer. Further, the dosage rate of each treatment is documented in **Table 1**. Briefly, CF (ratio 5:3:2) was applied three times as base fertilizer, as well as jointing and bell mouth stages. All commercial organic fertilizers, phosphate fertilizers, and nitrogen fertilizers were considered as base fertilizers.

### 2.3 Sampling and analysis

Soil samples were collected from the 0–5 cm, 5–10 cm, and 10–30 cm soil layers after maize harvest in 2019 and 2020 according to the “S” shape 5-point method and mixed evenly. After removing soil impurities such as plant residues and gravel, the soil samples were divided into two portions. One portion was sieved (2 mm) and stored at 4°C for the determination of MBC and DOC, and the other two portions were then air-dried and sieved through a 0.25 mm, stored for the determination of relevant soil physicochemical properties.

Determination of soil water content by using the oven method (Vachaud et al., 1985).

At corn maturity, soil capacity was determined using the ring knife method.

$\text{Soil porosity}=(1-\frac{\text{volumetric weight}}{\text{specific gravity}})*100\%$ , and soil specific gravity was 2.65 g·cm^−3^.

Soil pH was determined by the potentiometric method using a soil-water ratio of 1:2.5.

Soil total nitrogen was determined by H_2_SO_4_ boiling and the Kjeldahl method.

TP was determined by molybdenum antimony resistance colorimetric method. Available phosphorus was determined by molybdenum-antimony resistance colorimetric method. Nitrate-N and ammonium-N were determined by Smart Chem AST-6500S semi-automatic chemical discontinuous analyzer. All the chemical analysis methods were selected 166 according to Soil Agrochemical Analysis (Bao, 2000).

#### 2.3.1 Determination methods for soil carbon fractions

SOC was tested using the potassium dichromate oxidation method (Wen et al., 2013). To calculate SOC content, a soil sample of 15 mg was weighed and passed through a 0.25 mm sieve. Subsequently, the sample was transferred to a 50 ml centrifuge tube, into which 25 ml of a potassium permanganate solution with a concentration of 333 mmol/L was added and shaken for 1h. After centrifugation at 2,000 rpm for 5 min, the supernatant was diluted to a ratio of 1:250 with deionized water and transferred to a 250-ml volumetric bottle. The absorbance of the diluted sample was measured by a spectrophotometer at 565 nm, and a standard curve of potassium permanganate was prepared. EOC was analyzed by the KMnO_4_ oxidation method (Blair et al., 1995). Soil samples containing 15 mg of C were poured into 50 ml centrifuge tubes. Add 25 mL of potassium permanganate solution (333 mmol/L) and shake for 1h. Centrifuged, diluted, and shifted into a 250-ml volumetric flask, and then absorbance was measured at 565 nm by spectrophotometer.

To determine the LFOC, 25 g of soil was weighed and then placed in a 250-ml centrifugation tube. The centrifuge tube was subsequently filled with 50 ml of NaI solution with a specific gravity of 1.7 g/ml and manually shaken. Subsequently, the sample was subjected to agitation at 200 rpm for 1h and centrifugation at 3000 rpm for 20 min. Finally, the remaining fractions floating on the surface of the NaI solution were filtered by suction with 0.45 um nylon filter paper. After this, LFOC was left on the filter paper. The LFOC was washed several times with 0.0l mol/L CaC1_2_ solution; the apparatus was then rinsed with at least 75 ml of distilled water. Finally, the LFOC on the filter paper was transferred to a small beaker and placed for 1 day, dried in an oven at 60°C for 72h, weighed, and screened using a Vario MICRO cube elemental analyzer (Janzen et al., 1992). To analyze particulate organic carbon (POC), the air-dried soil sample of 20 g was put into 60 ml sodium hexametaphosphate (5 g/L), oscillated at 90 r/min for 18h, the suspension was passed through a 53-μm sieve, washed with pure water, and placed in a clean plate. The suspension was then dried at 60°C for 48h, and determined by a C automatic analyzer (Marriott and Wander, 2006). Furthermore, the concentration of dissolved organic carbon (DOC) was quantified by means of a cold water extraction method (Song et al., 2020). For this, DOC was extracted by 0.5 mol/L K_2_SO_4_ (water to soil ratio 4:1). After the extraction solution was filtered through a 0.45 µm-sized filter, and measured C by TOC analyzer. In addition, hot water-soluble carbon (HOC) was also calculated. To measure HOC, 10.000 of g soil was weighed and placed in a 150-ml wide-necked bottle. Fifty mililiter of deionized water was poured into the bottle, heated at 100°C for 1h, cooled, and remained there until the soil water content layer was clear and filtered. It should be noted that the supernatant was measured by a TOC analyzer (Ghani et al., 2003).

The MBC was estimated by the fumigation–extraction method (Vance et al., 1987). A 25 g of wet soil sample was weighed and placed in a culture cup. A portion of 12.5 g of soil (on an oven-dry basis) was subjected to a 24-h fumigation process at 25°C with ethanol-free CHCl_3_. Following the removal of the fumigant, the sample was extracted with 50 ml of 0.5 M K_2_SO_4_, shaken horizontally at 200 rpm for 30 min, and filtered through pleated filter paper. The second part was a non-fumigated soil sample of 12.5 g that was extracted. The extract was diluted 5–10 times with distilled water and measured MBC by TOC analyzer. The microbial biomass C was calculated in accordance with the following procedure:


MBC=EC/kEC,

Where EC = (organic C extracted from fumigated compost) – (organic C extracted from non-fumigated compost) and kEC is a constant factor with a value of 0.38.

#### 2.3.2 Crop yields and economic benefits

Following the harvesting and threshing of maize, the grains were separated and air-dried (maintaining a moisture content of 14%) in order to determine the grain yield (GY) per plot. Similarly, the biomass yield (BY) was also measured by 30 plants randomly selected from each plot. After all, the harvested data was converted from plot to per hectare yield. Further, the harvest index (HI) was calculated as follows:


HI=GY/BY

Where HI represents the HI, GY indicates the grain yield, and BY is the biomass yield.

A series of social surveys were conducted on local markets and agricultural outlets in order to assess and quantify the economic returns of each of the farming systems that were subjected to testing. The input items considered included labor costs, seed costs, plastic film costs, and organic biomass film costs. The principal output values were estimated on the basis of grain and straw yields. In this study, net income was calculated by subtracting the inputs from the output.

### 2.4 Data analysis

A statistical analysis was conducted utilizing the SPSS Statistics Software version 19.0. Analysis of variance was employed to assess variations in soil and plant different parameters. The treatment means were compared by computing the least significant difference in order to identify significant differences at the 0.05 probability level. Further, the Sigma Plot 12.5 was used to interpret study data in graphical form. The relationship between soil SOC and active organic carbon components and environmental factors (basic physical and chemical properties of soil) was analyzed with the Canoco 5.0 software redundancy analysis (RDA) module.

## 3 Results

### 3.1 Soil physical and chemical properties

**Figure 2:**
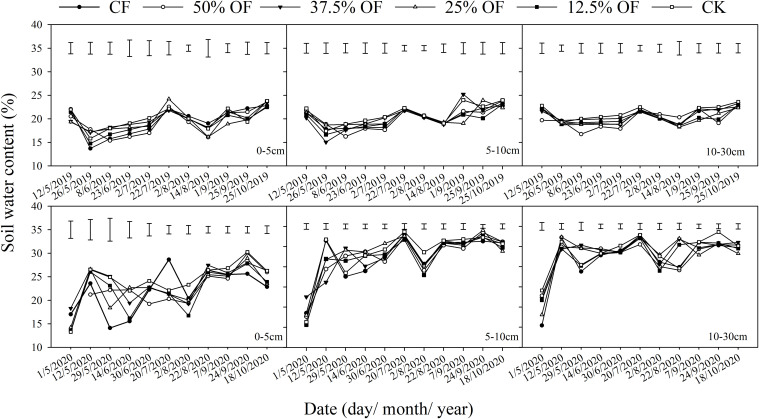
Change of soil water content in 0~30 cm under organic fertilizer replaces chemical fertilizer. The short vertical line at the top of the picture indicates the minimum standard error between different treatments at the same time.

At the seedling stage of maize (26/5/2019) (**Figure 2**), CF and 12.5% OF reduced the soil water content in the 0–5 cm soil layer by 13.35% and 6.64%, respectively, compared to CK. At the late filling stage (1/9/2019), all treatments reduced the moisture in the 5–10 cm soil layer by 9.89%–20.15%, except for 37.5% OF, which increased the soil water content compared to CK. At the grouting stage (25/9/2019), 50% OF and 12.5% OF reduced the soil water content in the 10–30 cm soil layer by 15.11% and 11.51% compared with CK, significantly. The soil water content in the 0–30 cm soil layer showed the following pattern: CK (20.96%) > 37.5% OF (20.57%) > CF (20.29%) > 25% OF (19.99%) > 12.5% OF (19.98%) > 50% OF (19.21%). At the sowing stage of 2020 (1/5/2020), CF and 25% OF treatments were reduced by 61.36% and 42.68%, respectively, compared to CK in 10–30 cm soil layer. At the flowering stage (2/8/2020), the soil water content was significantly reduced by 10.21%–26.81% and 13.90%–23.98% in the other treatments compared to CK, respectively. At the grouting stage (24/9), soil water content in the 0–5 cm and 10–30 cm soil layers of CF and 37.5% OF treatments decreased by 13.96%, 6.07%, and 11.55% and 10.74%, respectively, compared with that of CK. In the 0–30 cm soil layer showed the following results: CK (28.64%) > 25% OF (26.84%) > 50% OF (26.71%) > 12.5% OF (26.44%) > CF (25.52%) > 37.5% OF (24.78%), and CF and 37.5% OF were lower by 13.89% and 13.48%, respectively, than CK.

**Figure 3:**
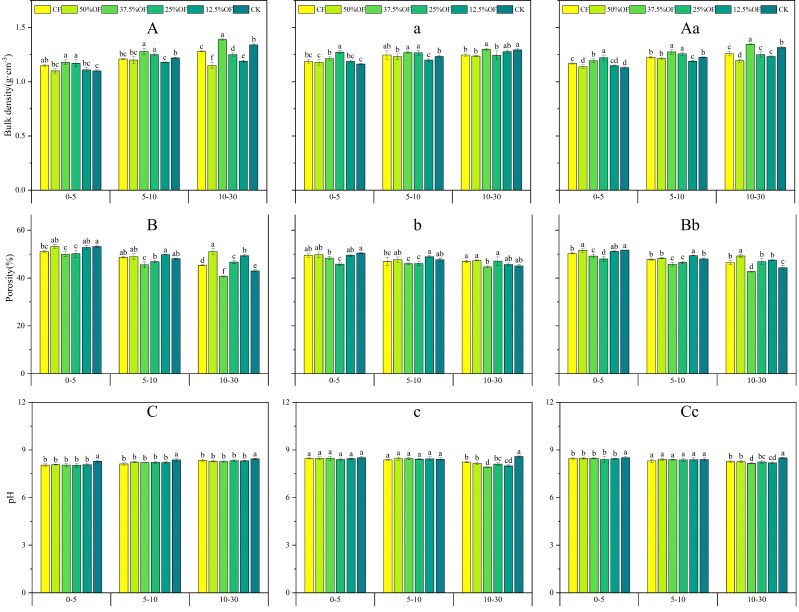
Changes of soil bulk density, porosity and pH under organic fertilizer replaces chemical fertilizer in 2019 **(A, B, C)**, 2020 **(a, b, c)** and two years average **(Aa, Bb, Cc)**. CF, 50%OF, 37.5%OF, 25%OF, 12.5%OF, and CK represent chemical fertilizer alone, organic fertilizer 50% substitution, organic fertilizer 37.5% substitution, organic fertilizer 25% substitution, and organic fertilizer 12.5% substitution for chemical fertilizer and no fertilization, respectively. Error bars indicate standard errors of the means (n = 3); different lowercase letters indicate significant differences between treatments at the 0.05 level.

Compared to CK, 25% OF significantly increased 0–5 cm capacity by 5.98%, 9.48%, and 8.18% in 2019, 2020, and 2 years average, respectively. 37.5% OF treatment significantly increased 10–30 cm soil layer capacity by 8.59%, 1.38%, and 7.2% in 2019, 2020, and 2 years average, compared to CF, respectively (**Figures 3A, a, Aa**). Soil porosity tended to decrease with soil layer deepening in 2019 and 2020. Compared with CK, 25% OF significantly reduced 0–5 cm porosity by 8.03%, 1.84%, and 2.31% in 2 years and its average, respectively. Fifty percent OF and 12.5% OF significantly increased by 47.66%–48.30% in 2019, 2020, and its average, compared with CK. 37.5% OF treatments was significantly reduced 10–30 cm soil layer porosity by 4.64%, 2.27%, and 3.74%, respectively, compared to CF (**Figures 3B, b, Bb**). Soil pH gradually increased with increasing soil depth. Compared with CK, the other treatments significantly reduced soil pH by 2.42%–3.02% in 0–5 cm over 2 years by 1.71%–3.20% (*P <* 0.05) in the 5–10 cm soil layer, and by 0.71%–1.78% in the 10–30 cm soil layer treatments, respectively (**Figures 3C, 3c, and 3Cc**).

**Figure 4:**
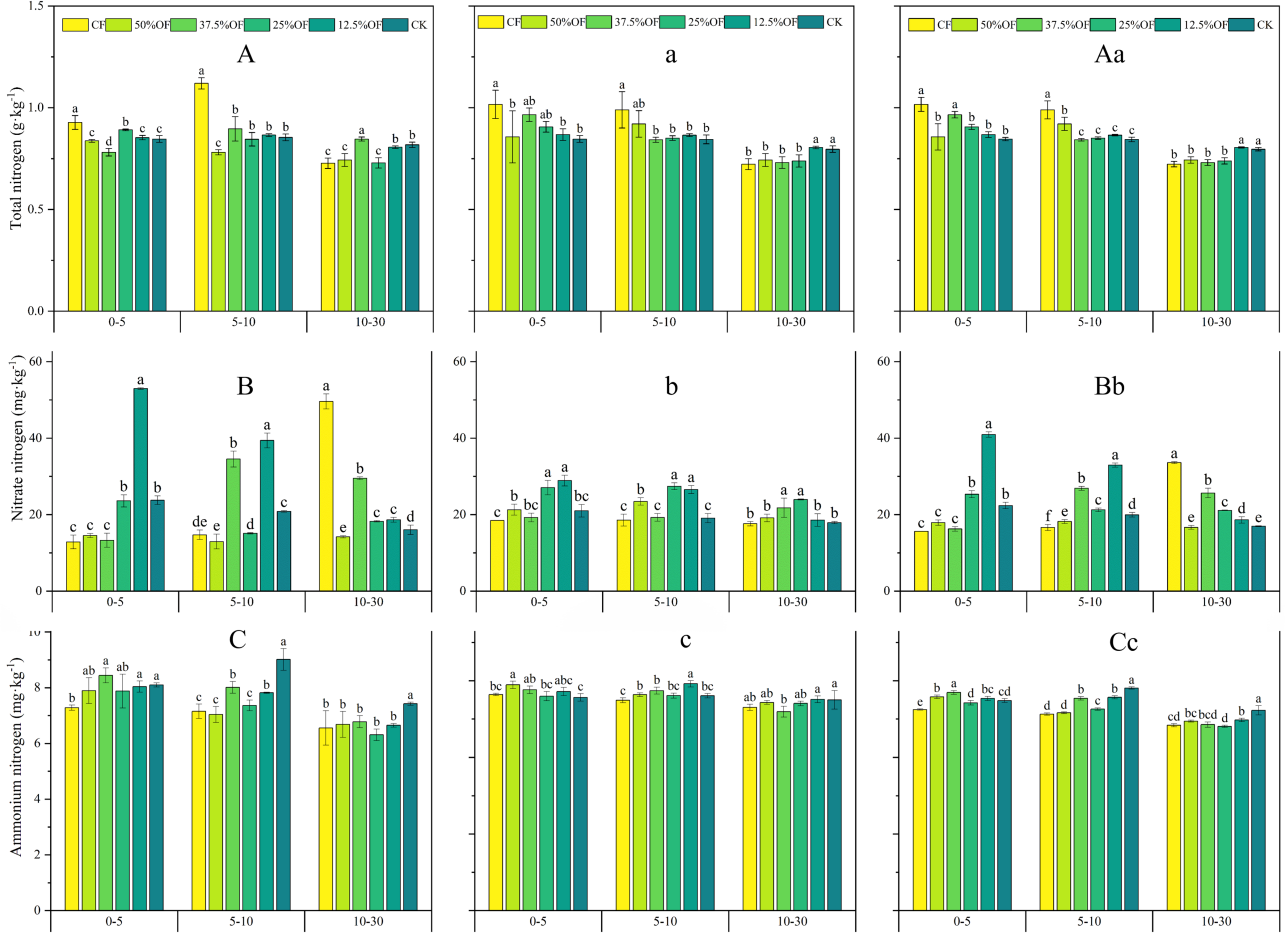
Changes of soil TN, NO_3_^-^ -N, NH_4_^+^ -N under organic fertilizer replaces chemical fertilizer in 2019 **(A, B, C)**, 2020 **(a, b, c)** and two years average **(Aa, Bb, Cc)**. Error bars indicate standard errors of the means (n = 3); different lowercase letters indicate significant differences between treatments at the 0.05 level. CF, 50%OF, 37.5%OF, 25%OF, 12.5%OF, and CK represent chemical fertilizer alone, organic fertilizer 50% substitution, organic fertilizer 37.5% substitution, organic fertilizer 25% substitution, and organic fertilizer 12.5% substitution for chemical fertilizer and no fertilization, respectively.

The TN content decreased as the soil layer deepened (**Figures 4A, 4a, and 4Aa**). In the 0–5 cm soil layer, CF was significantly higher by 4.49%–19.23% in 2019, 2020, and 2 years average, compared to the organic replacement treatments, and by 9.41%–20.13%, compared to CK. Soil NO_3_^–^N in the 0–5 cm soil layer was significantly increased by 3.26%–84.61%, 4.23%–56.55%, and 3.89%–161.33% in 2019, 2020, and 2 years average for other treatment, respectively, compared with CF. 25% OF and 12.5% OF treatments showed significant increase in soil NO_3_^–^N content in the 0–10 cm layer of the soil compared with CF and CK (**Figures 4B, b, Bb**). The NH_4_^+^-N content of each treatment decreased gradually with the increase of soil layers (**Figures 4C, c, Cc**). In 2019, in 0–5 cm soil layer, 37.5% OF increased significantly by 15.93% compared with CF; 50% OF increased significantly by 4.37% compared with CF in 2020. In addition, NH_4_^+^-N content of the soil layer of 5–30 cm showed that CK was higher than that of the organic replacement treatment, and the organic replacement treatment was higher than CF alone in 2020 and the 2 years average.

**Figure 5:**
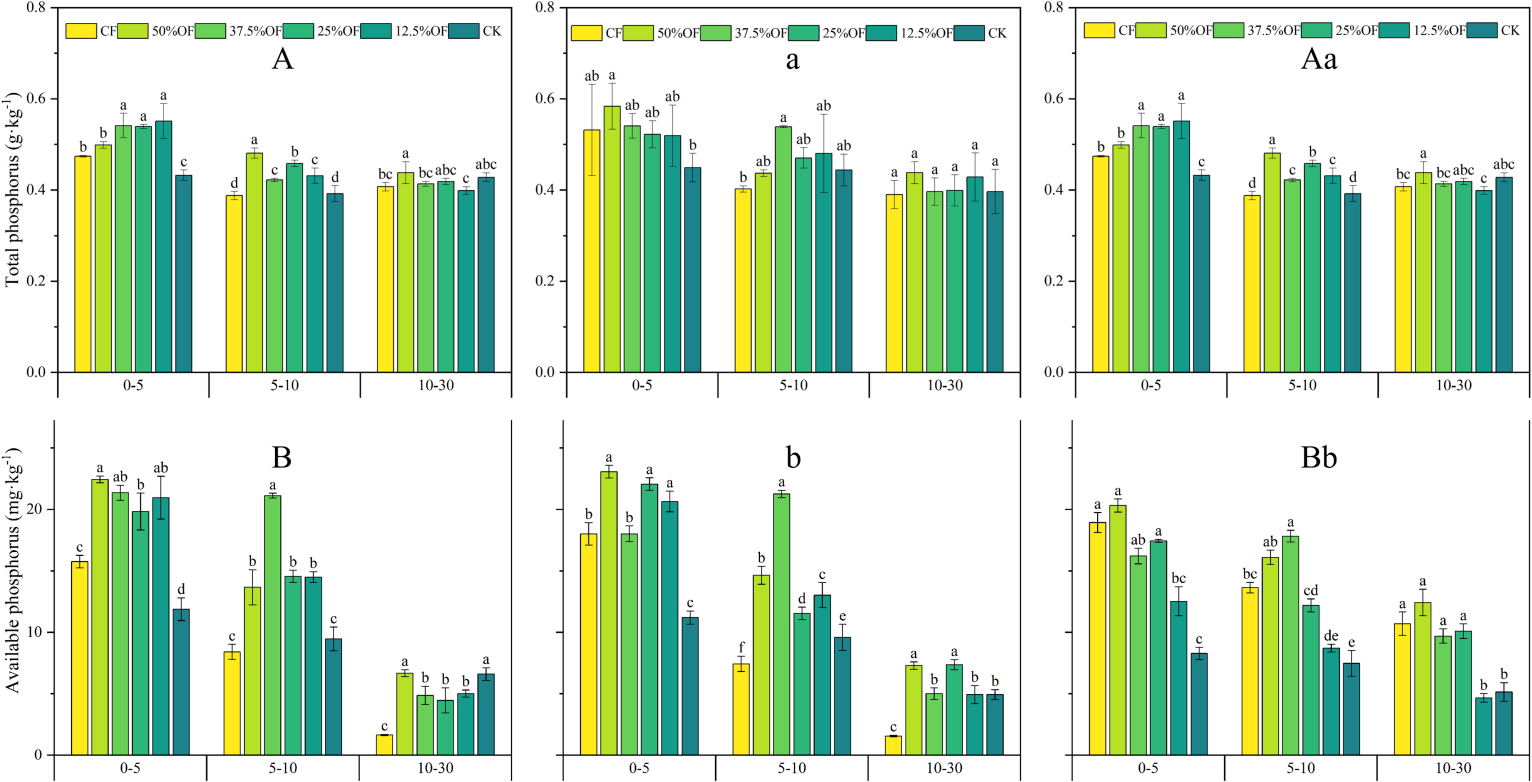
Changes of soil TP, AP under organic fertilizer replaces chemical fertilizer in 2019 **(A, B)**, 2020 **(a, b)** and two years average **(Aa, Bb)**. CF, 50%OF, 37.5%OF, 25%OF, 12.5%OF, and CK represent chemical fertilizer alone, organic fertilizer 50% substitution, organic fertilizer 37.5% substitution, organic fertilizer 25% substitution, and organic fertilizer 12.5% substitution for chemical fertilizer and no fertilization, respectively. Error bars indicate standard errors of the means (n = 3); different lowercase letters indicate significant differences between treatments at the 0.05 level.

Soil TP content decreased with deepening of the soil horizons (**Figures 5A, a, Aa**). In the 0–5 cm, 12.5% OF treatment significantly increased by 17.02% compared to CF and 27.91% compared to CK in 2019. In 5–10 cm soil layer, 12.5% OF was significantly increased by 13.16% compared to CF. In the 0–5 cm soil layer, the 2 years average organic replacement treatments increased soil TP content by 6.0%–8.0%. In the 5–10 cm soil layer, 12.5% OF significantly increased by 20.0% compared to CF. In the 5–10 cm soil layer. Fifty percent OF significantly increased by 10.26% compared to CF in the 10–30 cm soil layer. Soil AP decreased with deeper soil layers (**Figures 5B, b, Bb**). In the 0–5 cm soil layer, CF significantly decreased by 16.58%–42.47%, 14.60%–28.09%, and 16.58%–34.77%, respectively, compared with other treatments in 2019, 2020, and 2 years average. In the 5–10 cm soil layer, CF significantly decreased by 62.5%–151.42% compared with other treatments. In addition, in the 10–30 cm soil layer, CF was significantly reduced by 306.71%, 196.34%, 171.34%, and 206.10% in 2019 compared to the organic replacement treatments. In 2020, CF was significantly reduced by 372.72%, 224.03%, 377.27%, and 219.48%, respectively, compared to the organic replacement treatments.

### 3.2 SOC

**Figure 6:**
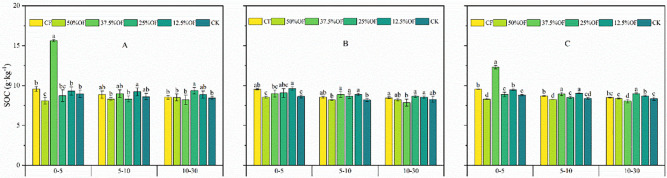
Changes of SOC content under organic fertilizer replaces chemical fertilizer in 2019 **(A)**, 2020 **(B)** and two years average **(C)**. CF, 50%OF, 37.5%OF, 25%OF, 12.5%OF, and CK represent chemical fertilizer alone, organic fertilizer 50% substitution, organic fertilizer 37.5% substitution, organic fertilizer 25% substitution, and organic fertilizer 12.5% substitution for chemical fertilizer and no fertilization, respectively. Error bars indicate standard errors of the means (n = 3); different lowercase letters indicate significant differences between treatments at the 0.05 level.

The SOC content of the treatments in the 0–30 cm soil layer decreased with increasing soil layer (**Figure 6**). In the 0–5 cm soil layer, 37.5% OF treatment significantly increased SOC content by 63.39% and 28.82% compared to CF in 2019 and 2 years average. In the 10–30 cm soil layer, the 25% OF treatment significantly increased 9.74% and 6.0% compared to CF in 2019 and the 2 years average, respectively. In 2019, the 37.5% OF, 25% OF, and 12.5% OF treatments significantly increased by 9.37%, 3.77%, and 2.86%, respectively, compared to CF in the 0–30 cm soil layer. In 2020, in the 0–30 cm soil layer, 12.5% OF and 25% OF were significantly higher by 1.50% and 0.81%, respectively, compared to CF.

### 3.3 Soil carbon fractions

**Figure 7:**
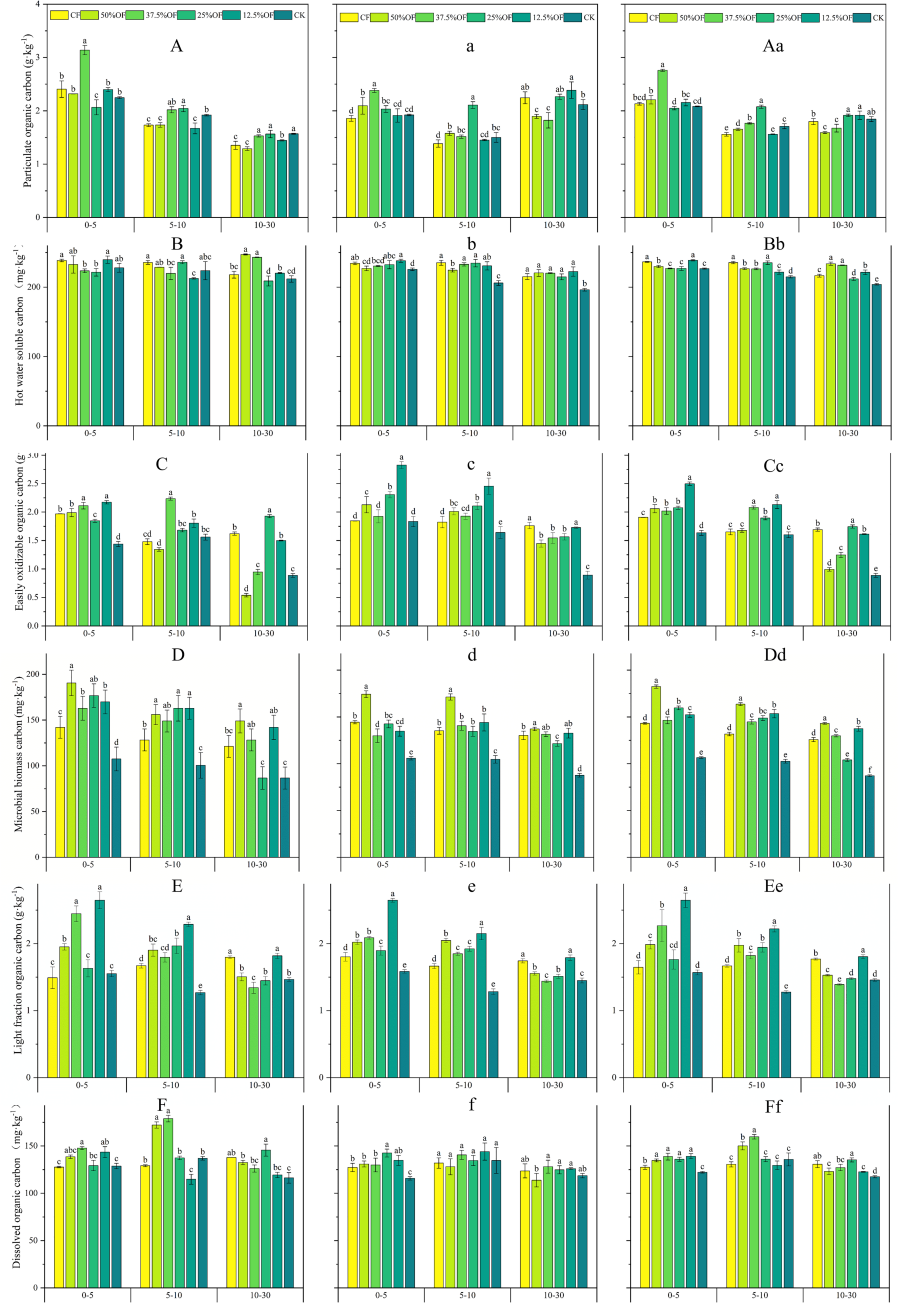
Changes of SOC fractions content under organic fertilizer replaces chemical fertilizer in 2019 **(A, B, C, D, E, F)**, 2020 **(a, b, c, d, e, f)** and two years average **(Aa, Bb, Cc, De, Ee, Ff)**. CF, 50%OF, 37.5%OF, 25%OF, 12.5%OF, and CK represent chemical fertilizer alone, organic fertilizer 50% substitution, organic fertilizer 37.5% substitution, organic fertilizer 25% substitution, and organic fertilizer 12.5% substitution for chemical fertilizer and no fertilization, respectively. Error bars indicate standard errors of the means (n = 3); different lowercase letters indicate significant differences between treatments at the 0.05 level.

As shown in **Figure 7**, in the 0–5 cm soil layer, the EOC content of the 12.5% OF treatment was significantly increased by 13.20% and 53.26% (*P* < 0.05) in 2019 and 2020, respectively, compared to CF. In the 0–30 cm soil layer, the EOC content of the 12.5% OF treatment was increased by 13.94%, 13.33%, and 6.97%, respectively, compared to CF. DOC content was 9.11% (*P* < 0.05) higher in 12.5% OF treatment compared to CF in 0–5 cm soil layer. Organic replacement treatments were significantly higher than CK (4.28%–11.66%). In the 0–5 cm soil layer, the 12.5% OF treatment increased by 70.22%, 67.08%, and 69.23% (*P* < 0.05) compared to CK in 2019, 2020, and 2 years average, respectively. In the 5–10 cm soil layer, the 12.5% OF treatment increased by 37.13%, 67.96%, and 74.01% compared to CK.

POC content decreased gradually with deepening of the soil layer. In the 0–5 cm soil layer, 37.5% OF increased POC content by 30.29%, 23.95%, and 32.21% over CF in 2019, 2020, and 2 years average, respectively. The 2 years average was a significant increase of 6.70% and 6.64% for 25% OF and 12.5% OF treatments compared to CF, respectively. In the 10–30 cm soil layer, the HOC content was 13.20% and 6.90% higher (*P* < 0.05) for the 37.5% OF treatment than the CF treatment in 2019 and the 2 years average, respectively. The average HOC of all fertilized treatments was higher than CK in both years, ranging from 10.62% to 44.11%. MBC content decreased with increasing soil layer. In 0–5 cm soil layer, 50% OF treatment was significantly higher than CF and CK by 21.37%–77.41% in 2019, 2020, and on average. In 5–10 cm soil layer, 2019, 2020, and average, 12.5% OF treatment significantly increased 26.63%–87.99% compared to CK and −0.45%–27.03% compared to CF. In 10–30 cm soil layer, 12.5% OF treatment was 48.00%–51.32% higher than CK. 50% OF and 12.5% OF treatments were 64.64% and 53.31% higher than CK and 18.03% and 9.90% higher than CF, respectively.

### 3.4 Maize yield

Data in **Table 2** shows that different treatments have significant effects on maize grain yield and biological yield. The grain yield in 2019 was 13.65% higher than that in 2020, and the biological yield was 7.22% higher than that in 2020. In 2019 and 2020, the grain yield of four treatments was similar to CF, but it was higher than CK, and the increase rates in the 2 years were 146.77%–290.65% and 107.28%–148.16%, respectively. In addition, the grain yield and biological yield under 12.5% OF treatment increased by 6.60% and 4.59% compared with CK (*P* > 0.05) and increased by 38.96% and 20.72% compared with those at 50% OF treatment (*P* < 0.05). In 2019 and 2020, the yield of each replacement level compared with CK significantly increased by 208.52%–343.63% and 115.90%–144.30%, respectively. The HI remained unchanged across all treatments in both years.

**Table 2:** Effects of organic fertilizer replaces chemical fertilizer on maize biomass accumulation, grain yield and harvest index (HI).

Treatment	Grain yield (kg·ha^-1^)		Biomass (kg·ha^-1^)		Harvest Index	
	2019	2020	2019	2020	2019	2020
CF	10100ab	8396ab	37641a	35749a	0.28a	0.24a
50%OF	7075b	7114b	31059a	32524a	0.24a	0.22a
37.5%OF	9767ab	8416ab	39263a	36264a	0.25a	0.24a
25%OF	8703ab	7865ab	34097a	32048a	0.26a	0.25a
12.5%OF	11200a	8517a	44660a	32099a	0.25a	0.27a
CK	2867c	3432c	10067b	14844b	0.30a	0.24a

Error bars indicate standard errors of the means (n = 3); different lowercase letters indicate significant differences between treatments at the 0.05 level. CF, 50%OF, 37.5%OF, 25%OF, 12.5%OF, and CK represent chemical fertilizer alone, organic fertilizer 50% substitution, organic fertilizer 37.5% substitution, organic fertilizer 25% substitution, and organic fertilizer 12.5% substitution for chemical fertilizer and no fertilization, respectively.

### 3.5 Economic benefit

As illustrated in **Table 3**, there are discrepancies in the quantity of CF and commercial organic fertilizer inputs, as well as in the total input of different treatments is also different. The total input of CF and 12.5% OF treatments increased by 2120, 5885, 944, 4002, and 3062 yuan*ha^−1^, respectively. The total output values of fertilization treatments were higher than CK in 2019. The 37.5% OF and 12.5% OF treatments were equivalent to CF, while the 50% OF and 25% OF treatments were 22.6% and 11.2% lower than CF, respectively. The output-to-input ratio for these treatments was lower than CF but higher than CK. In 2020, except that the total output value of 37.5% OF treatment was equivalent to CF, the total output value of other substitution levels was lower than CF, but higher than CK, increasing by 114.7%–128.99%. The yield-input ratio of CF exceeded that partial substitution of CF with organic fertilizer, and the yield-input ratio of an organic replacement treatment was higher than non-fertilization. In 2 years, the total output values of CF and 12.5% OF treatments were 30,371 and 32,006 yuan ha^−1^, respectively, and the yield-input ratios were 2.33 and 2.29, respectively. From the above result mentioned in **Table 3**, 12.5% OF treatment can achieve the same effect as CF in 2 years.

**Table 3:** Effect of organic fertilizer replacing chemical fertilizer on economic benefit of maize.

Year	Treatment	Input yuan ha^-1^	Total input Yuan·ha^-1^	Output yuan ha^-1^		Gross output Yuan·ha^-1^	Input-output ratio
		Fertilizer		Straw	Grain		
2019	CF	2120	13025	13771	18180	31951	2.45
	50%OF	5885	16790	11992	12735	24727	1.47
	37.5%OF	4944	15849	14748	17581	32329	2.04
	25%OF	4022	14907	12697	15666	28363	1.9
	12.5%OF	3062	13967	16730	20160	36890	2.64
	CK	0	10905	3600	5161	8761	0.8
2020	CF	2120	13025	13677	15113	28790	2.21
	50%OF	5885	16790	12705	12805	25510	1.52
	37.5%OF	4944	15849	13924	15149	29073	1.83
	25%OF	4022	14907	12092	14157	26249	1.76
	12.5%OF	3062	13967	11791	15331	27122	1.94
	CK	0	10905	5706	6178	11884	1.09

The prices of seed, plastic film, labor, and machinery were 720, 2160, 7,425, and 600 yuan ha^-2^, respectively. The input of labor, including the whole labor input of human, animal, etc., the price was 60 yuan d^-1^; the grain price accorded to the average price in the test area, as grain 1.8 yuan kg^-1^ and straw 0.5 yuan kg^-1^, respectively.

### 3.6 Relationship between maize yield and soil carbon fractions

#### 3.6.1 SOC, labile organic carbon fractions, and environmental factors

**Figure 8:**
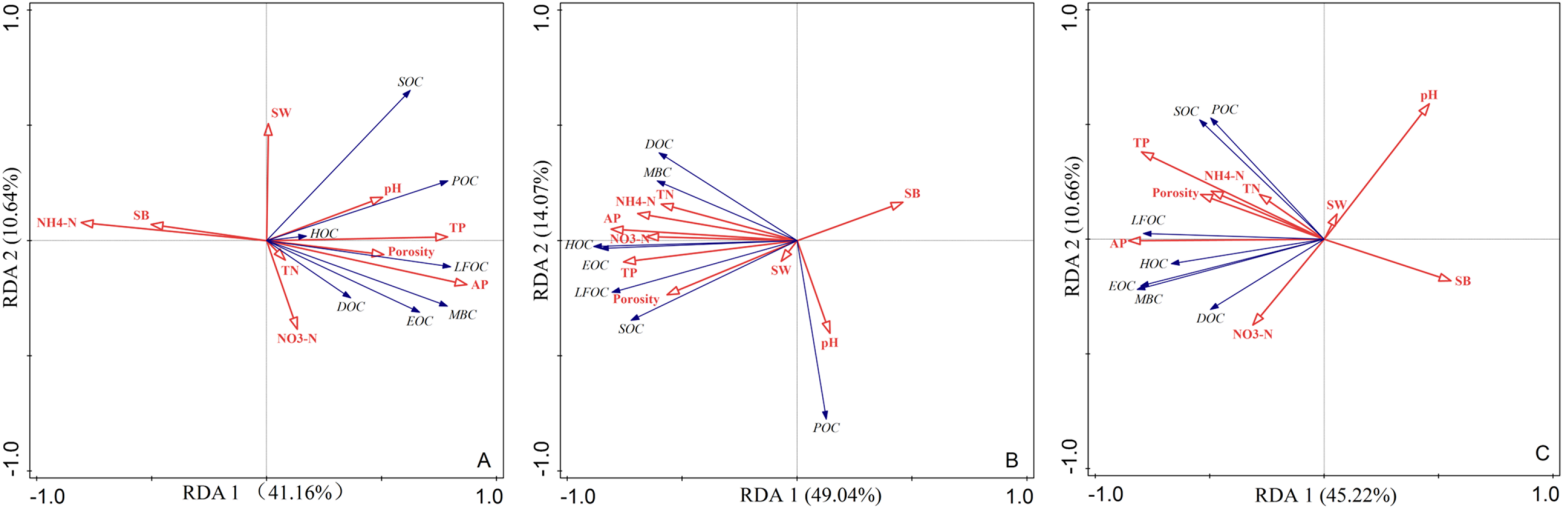
Redundancy analysis (RDA) of SOC, labile organic carbon fractions, and environmental factors in 2019 **(A)**, 2020 **(B)** and two years average (C). SOC and fractions are indicated by black lines, and environmental factors are indicated by red lines.

The results of RDA analysis of soil SOC and reactive organic carbon fractions with environmental factors (**Figure 8**). In 2 years and 2 years average, environmental factors explained 51.8%, 63.11%, and 55.88% of soil SOC and reactive SOC fractions, respectively. The first ordering axes explained 41.16%, 49.04%, and 45.22% of this variation, while the second ordering axes contributed additional explanatory power. In 2019, NH_4_^+^-N, soil TP, and soil AP were positively correlated with SOC and carbon fractions, while negatively correlated with SB, and positively correlated with SOC fractions. In 2020, SOC and active organic carbon fractions were positively correlated with soil NO_3_^–^N, soil TP, soil AP, and soil porosity, and positively correlated with soil pore size. In 2020, SOC and active organic carbon components were positively correlated with soil NO_3_^−^-N, soil TP, soil AP, and soil porosity, and negatively correlated with SB and pH.

#### 3.6.2 SOC, labile organic carbon fractions, and maize yield

**Figure 9:**
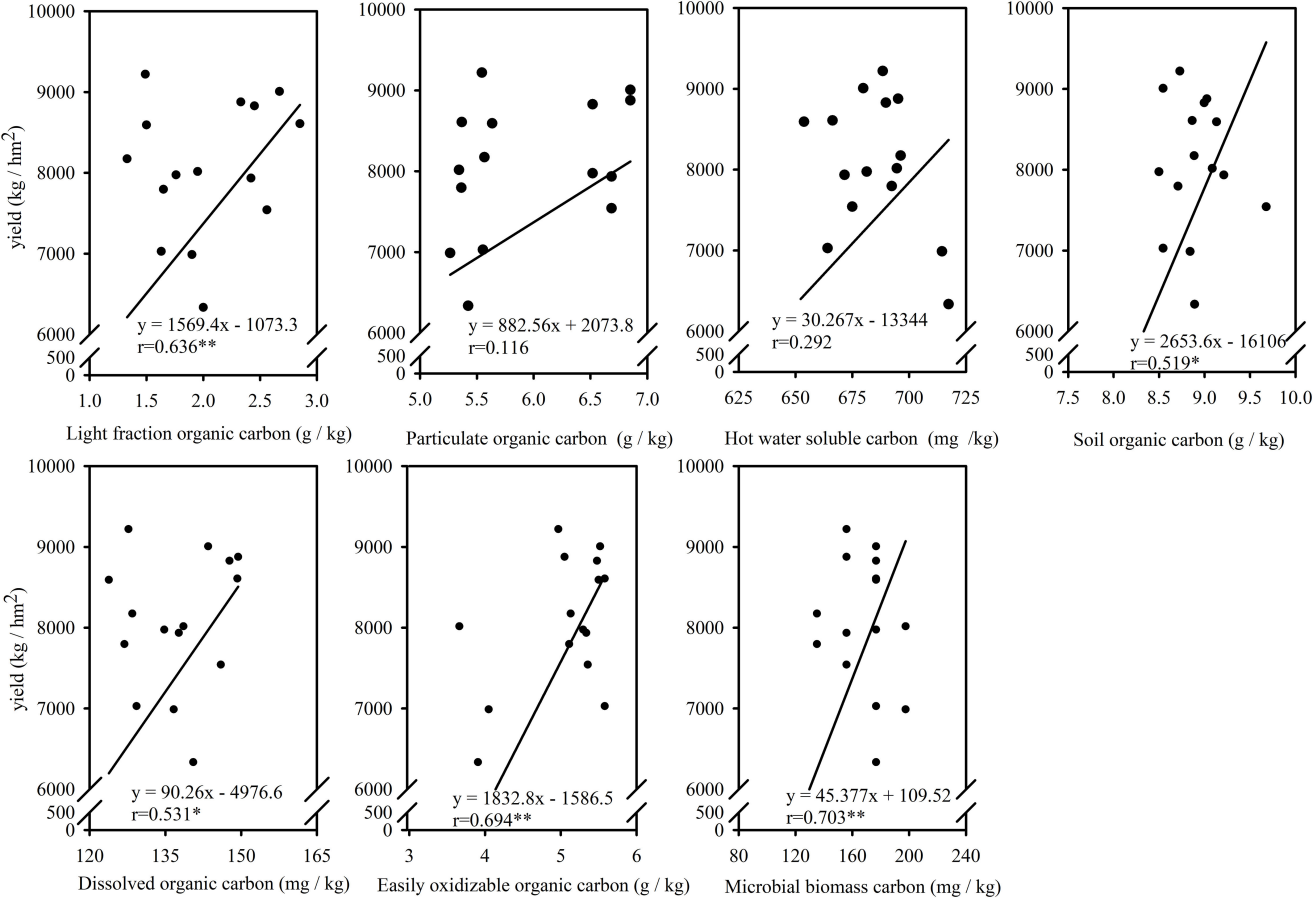
Correlate relationships of maize yield and different soil carbon fraction. *, significant difference (*P<0.05*), **, extremely significant difference (*P<0.01*).

As shown in **Figure 9**, the correlation analysis between maize yield and SOC demonstrated a significant positive correlation between the two variables. A significant positive correlation was observed between maize yield and SOC, as well as SOC fractions. In addition, there was a significant positive correlation between soil LFOC, soil EOC, and soil MBC and maize yield. There was a significant correlation between soil DOC content and maize yield; these results indicated that soil LOFC, soil EOC, soil MBC, and soil DOC were closely related to maize yield. Therefore, maize yield can be further improved by increasing soil active organic C content such as LFOC, DOC, EOC, and MBC.

**Figure 10:**
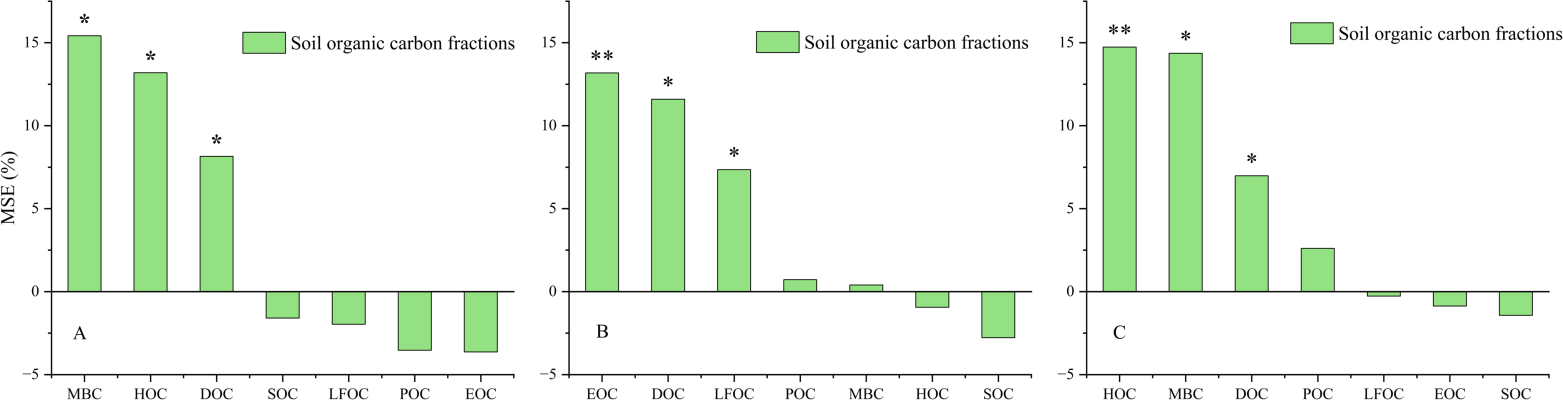
The predictors of maize yield based on random forest under different organic fertilizer substitutions in 2019 **(A)**, 2020 **(B)** and two years average **(C)**. SOC, Soil organic carbon; MBC, Microbial biomass carbon; HOC, Hot water soluble carbon; DOC, Dissolved organic carbon; EOC, Easily oxidizable organic carbon; POC ,Particulate organic carbon; LFOC, light fraction organic carbon. *, significant difference (*P<0.05*), **, extremely significant difference (*P<0.01*).

Random forest algorithm determined the relationship between maize yield, SOC, and fractions (**Figure 10**). In this study, it was found that soil MBC, HOC, and DOC reached significant levels of 15.41%, 13.19%, and 8.15%, respectively, in 2019. In 2020, soil EOC reached highly significant level, DOC, and LFOC reached significant levels of 13.17%, 11.59%, and 7.35%, respectively. The two-year average is soil HOC reached a highly significant level, MBC, and DOC reached a significant level with 14.73%, 14.36%, and 6.98%, respectively.

## 4 Discussion

### 4.1 Organic fertilizer substitution improves soil properties and thus SOC

Soil physical and chemical properties are the main indicators to evaluate the soil quality (Feng et al., 2024). The dynamics of different organic and inorganic carbon pools can indirectly reflect the mechanism of partial substitution of CF with organic fertilizer on soil quality (McLauchlan and Hobbie, 2004). Partial substitution of CF with organic fertilizer increases soil porosity and SWC, accelerates organic matter mineralization, speeds up nutrients release, and increased SOC in farmland ecosystems (Xu et al., 2023). In this study, SB was significantly negatively correlated with SOC and carbon fractions in 2019. Soil porosity was significantly and positively correlated with SOC and carbon fractions. Another study showed that organic fertilizers boost soil NH_4_-N, NO_3_-N, TP and AP contents while raising SB and lowering pH (Bahadori et al., 2021a). Similar results were found in the present study. In 2020, 12.5% OF significantly enhanced NO_3_^–^N, TN, NH_4_^+^-N and AP in 0–30 cm soil compared with CF. NO_3_^–^N and NH_4_^+^-N were positively correlated with SOC and carbon fractions. Many studies have found that soil pH was significantly positively correlated with SOC content and negatively correlated with soil NO_3_^−^-N content in trials where partial substitution of CF with organic fertilizer (Song et al., 2022; Xu et al., 2023). The same results were obtained in our study. This study showed that soil TN, TP, NH_4_^+^-N, NO_3_^–^N, pH, and porosity were significantly correlated with soil carbon fractions. This finding is consistent with the results of existing studies that organic fertilizers can ultimately promote the increase of SOC by decreasing SB, increasing soil porosity, enhancing soil water-holding capacity, regulating soil pH, and increasing soil TN and TP content (Stewart et al., 2008; Wan et al., 2024). Studies have shown that high nitrogen environments accelerate the rate of decomposition of early apoplastic litter and increase the proportion of degradable components in the litter. This process not only directly increases SOC content but also provides more attachment sites for soil microorganisms. Microorganisms further contribute to the input of SOC by chemically binding to the plant apoplast (Tang et al., 2023). This increases the exogenous carbon input to the soil and at the same time increases the TN content of the soil. Most studies have concluded that the application of organic fertilizers instead of CFs can increase the storage and utilization of N in the soil and increase the TN content of the soil by improving the soil structure and increasing the soil microbial population (Dai et al., 2021; Liu et al., 2022; Song et al., 2022). The partial substitution of CF with organic fertilizer in our study reduced soil surface TN, probably because the addition of organic fertilizers reduced the excessive use of nitrogen fertilizers, which in turn reduced TN. Further, it has been shown that partial substitution of CF with organic fertilizer promoted greater accumulation of free and confined POC in chestnut laurel soils (Ouédraogo et al., 2006). This is consistent with our study that partial substitution of CF with organic fertilizer effectively prevented the reduction of POC content in soil compared to using CF alone. Some studies have discovered that the interactive effect of organic fertilizer and CF was remarkable on EOC as compared without organic fertilizer (Yang et al., 2015). The TN and TP contents of the soil were positively correlated with the EOC content, while pH and SB were negatively correlated with the EOC (Wang et al., 2020). In this study, fertilization treatments increased TN and TP content and decreased pH, which in turn promoted decomposition and transformation of organic matter and increased EOC content. Our study confirmed by the fact that fertilizer treatment increased EOC by 24.77%–72.48% compared with no fertilizer treatment in this study. Partial substitution of CF with organic fertilizer may enhance soil C and N, alleviate microbial competition for N, and enrich the carbon source of soil microorganisms (Wu et al., 2022). Nutrient levels such as nitrogen and phosphorus in the soil are critical for microbial growth. In this study, soil total nitrogen and phosphorus content had significant positive correlation with MBC. This in agreement similar findings were also observed as they reported that changes in nutrient content affected the soil MBC content (Jia et al., 2020). Some studies investigated that farmyard manure significantly increased DOC and improved soil quality compared to no fertilization (Simon and Czakó, 2014). The present study exhibited that organic treatments (50% OF, 37.5% OF, and 12.5% OF) significantly increased 0–30 cm DOC content compared to CK and their DOC content was not lower than that of CK. In the forest floor on the Loess Plateau of China, soil pH, TN, and TP affect microbial metabolic activity and the rate of decomposition of organic matter, which in turn affects DOC (Wang et al., 2020; Bahadori et al., 2021b). In this study, the decrease of pH, the increase of TN and TP favored the increase of DOC content. Moreover, soil LFOC is one of the active organic C fractions and an important index for measuring SOC. Studies have shown that high soil moisture content can further stimulate the development of plant roots and crops, increase crop litter, and further increase soil LFOC content (Xu et al., 2023). Currently, we have observed that soil water may lead to an increase in soil LFOC content. In addition, some studies found that soil water did not affect soil LFOC, which might be related to different soil hydrothermal conditions (Christensen, 1992). Previously, it is stated that fertilization affects soil C cycle, which ultimately changes the HOC content in soil (Xie et al., 2007; Gao et al., 2016). At the 10–30 cm soil layer, 50% OF and 37.5% OF treatments significantly increased HOC content 13.25%, 13.20%, 13.36%, and 11.50%, respectively compared with CF or CK in 2019. In summary, the improvement of soil physicochemical properties by partial substitution of CF with organic fertilizer is an important reason for the improvement of SOC.

### 4.2 Increased soil SOC ensures crop yields and economic benefits

Previous studies have documented that labile organic carbon fractions can enhance yield by regulating soil fertility (Chathurika et al., 2019). Similar patterns were found in the present study, with a significant positive correlation between soil C components (i.e., SOC, MBC, LFOC, EOC, and DOC) and maize yield. This suggests that C is an important energy source to promote soil microbial activities and crop yield (Demisie et al., 2014). It has been demonstrated a significant positive correlation between crop yield and MBC (Song et al., 2017). Dang et al. (2015) have found that applying organic fertilizer alone or combined with CF significantly enhanced spring maize yield compared to no fertilizer. In this study, replacing inorganic N and organic N notably improved maize grain and biological yields. This, due to higher organic fertilizer application, improves crop C and N nutrition, resulting in maize yields at four substitution levels being comparable to CF, ensuring stable productivity (Zhou et al., 2023). Studies have found that organic fertilizer combined with chemical fertilizer meets the needs of maize growth in the early stage (Xie et al., 2019). Additionally, plays a slow-release role in the later stage, and provide sustained C and N nutrients. This mechanism ultimately increases maize yield (Efthimiadou et al., 2010). Our study demonstrated that the grain yield of the control decreased significantly, which may be due to fact that this experiment was a long-term positioning experiment, where continuous planting of maize without fertilizer would lead to soil nutrient depletion and insufficient nitrogen supply for maize growth (Liu et al., 2021). Additionally, the grain yield at 50% OF treatment was 28.04% lower than that at 12.5% OF treatment. Also, this could be due to the continuous CF input level of 200 kg ha^−1^, a moderate reduction in the proportion of organic fertilizer can obtain a higher yield level (Zhang et al., 2018; Xiao et al., 2016). Studies by Xu et al. (2021) have indicated that higher application rates benefited crop yields when the same commercial organic fertilizer was applied. Some studies have indicated that the application of organic fertilizer in moderation can enhance the yield of cereal crops such as millet by improving the ear weight, and thousand-grain weight (Gao et al., 2024). The application of organic fertilizer increases input, when combined with CF, whereas reasonable ratio of N, P, and K is beneficial for improving crop nutrition accumulation (Ferdous et al., 2020). This suggests the same effect as CF in terms of yield and output–input ratio, with better economic benefits. In addition, the HI of each treatment was low in the 2 years, which may be a wet year. The 12.5% OF and 37.5% OF in the full film double-ridge sowing system effectively increased soil SOC content, which in turn ensured maize yield and economic benefits (Xu et al., 2023).

## 5 Conclusion

This study showed that the 12.5% OF and 37.5% OF treatments increased soil porosity and soil TP and effective phosphorus (AP) content, while reducing water consumption, thereby optimizing the soil’s ability to retain water and fertilizer. This further increased soil MBC, hot water-soluble carbon (HOC), and DOC content, laying the foundation for high crop yields. This provides a theoretical basis for partial substitution of CF with organic fertilizer to improve soil health and crop yield. Compared to 37.5% OF, 12.5% OF is more environmentally sustainable and is recommended for wider use in the region.

## Data Availability

The original contributions presented in the study are included in the article/supplementary material. Further inquiries can be directed to the corresponding author.
